# Juglone from Walnut Produces Cardioprotective Effects against Isoproterenol-Induced Myocardial Injury in SD Rats

**DOI:** 10.3390/cimb44070220

**Published:** 2022-07-16

**Authors:** Taseer Ahmad, Taous Khan, Tahira Tabassum, Yahya S. Alqahtani, Mater H. Mahnashi, Bandar A. Alyami, Ali O. Alqarni, Mohammed Y. Alasmary, Sultan A. Almedhesh, Abdul Jabbar Shah

**Affiliations:** 1 Department of Pharmacy, COMSATS University Islamabad, Abbottabad Campus, University Road, Abbottabad 22060, Pakistan; taseer.ahmad@uos.edu.pk (T.A.); taouskhan@cuiatd.edu.pk (T.K.); 2 Laboratory of Cardiovascular Research and Integrative Pharmacology, College of Pharmacy, University of Sargodha, Sargodha 40100, Pakistan; 3 Department Pathology, Sargodha Medical College, University of Sargodha, Sargodha 40100, Pakistan; tahira.tabassum@uos.edu.pk; 4 Department of Pharmaceutical Chemistry, College of Pharmacy, Najran University, Najran 61441, Saudi Arabia; yahyasalqahtani0@gmail.com (Y.S.A.); matermaha@gmail.com (M.H.M.); alyamibandar1@gmail.com (B.A.A.); aoqarni@gmail.com (A.O.A.); 5 Medical Department, College of Medicine, Najran University, Najran 61441, Saudi Arabia; myalasmary@nu.edu.sa; 6 Pediatric Department, College of Medicine, Najran University, Najran 61441, Saudi Arabia; almedhesh31@hotmail.com

**Keywords:** myocardial infarction (MI), juglone, antioxidant, isoproterenol, ECG, cardiac marker enzymes, histopathology

## Abstract

Therapeutic and/or preventive interventions using phytochemical constituents for ischemic heart disease have gained considerable attention worldwide, mainly due to their antioxidant activity. This study investigated the cardioprotective effect and possible mechanism of juglone, a major constituent of the walnut tree, using an isoproterenol (ISO)-induced myocardial infarction (MI) model in rats. Rats were pretreated for five (5) days with juglone (1, 3 mg/kg, i.p) and atenolol (1 mg/kg, i.p) in separate experiments before inducing myocardial injury by administration of ISO (80 mg/kg, s.c) at an interval of 24 h for 2 consecutive days (4th and 5th day). The cardioprotective effect of juglone was confirmed through a lead II electrocardiograph (ECG), cardiac biomarkers (cTnI, CPK, CK-MB, LDH, ALT and AST) and histopathological study. The results of our present study suggest that prior administration of juglone (1 and 3 mg/kg) proved to be effective as a cardioprotective therapeutic agent in reducing the extent of myocardial damage (induced by ISO) by fortifying the myocardial cell membrane, preventing elevated T-waves, deep Q-waves in the ECG, heart to body weight ratio, infarction and also by normalizing cardiac marker enzymes (cTnI, CPK, CK-MB, LDH, ALT and AST) and histopathological changes, such as inflammation, edema and necrosis. In conclusion, this study has identified phytochemical constituents, in particular juglone, as a potential cardioprotective agent.

## 1. Introduction

Myocardial ischemia or cardiac ischemia is characterized by an imbalance between myocardial oxygen supply and demand, causing cardiac dysfunction, arrhythmias, MI, and sudden death [1]. MI is defined as myocardial cell death due to prolonged ischemia [2]. There are approximately 32.4 million MI and strokes cases that are reported globally every year [3]. The overall prevalence of IHD in Pakistan is 26.9% [4]. More than 30% of the population over 45 years of age is affected by MI [5]. MI is invariably followed by numerous pathophysiological and biochemical alterations, such as hyperlipidemia, thrombosis, lipid peroxidation (LPO), free radical damage and decline in nitric oxide (NO) levels, leading to qualitative and quantitative changes in the myocardium [6]. The heart is one of the major organs affected by reactive oxygen species (ROS) and oxidative stress [7]. It has also been suggested that oxidative stress produced by free radicals (including its type ROS), as evidenced by a marked increase in the production of lipid peroxidative products associated with decreased levels of antioxidants’ defense system, such as superoxide dismutase (SOD), catalase (CAT) and reduced glutathione peroxidase (GPx), plays a major role in myocardial damage during MI [8]. MI is also linked with the pathological alterations due to atherosclerosis. Atherosclerosis is by far the most common cause of MI [9]. In addition, pathophysiological alterations also include inhibition of NOS activity [10], over expression of calcium channels [11], cytosolic Ca^2+^ overload [12], blockade of potassium channels [10] and elevated cardiac markers, including cardiac troponin I (cTnI), creatine phosphokinase (CPK), creatine kinase-myocardial band (CK-MB), lactate dehydrogenase (LDH), alanine aminotransferase (ALT) and aspartate aminotransferase (AST) [13,14,15]. A gold standard marker for the diagnosis of MI is cardiac troponins (cTn) [16]. 

Cardioprotection includes all mechanisms and means that contribute to the preservation of the heart by decreasing or even preventing myocardial damage [17]. The principal pharmacological agents used in the treatment of MI are nitrovasodilators (nitroglycerine), β adrenergic receptor antagonists (atenolol) and Ca^2+^ channel antagonists (verapamil). However, some limitations exist, such as nitroglycerin and nitrates can cause vasodilation-induced headaches, adverse effects of beta-blockers include bradycardia, bronchoconstriction, sexual dysfunction and hypoglycemia and calcium channel blockers are potent vasodilators that lead to excessive hypotension, and reflex tachycardia [18]. In order to search for new cardioprotective agents, the extracts and phytochemical constituents of many medicinal plants have been investigated and reported to possess cardioprotective effects mediated through different mechanisms [19,20]. Natural products including phenolic compounds have been used as the basis of treatment of ischemic heart diseases [21,22].

**Figure 1:**
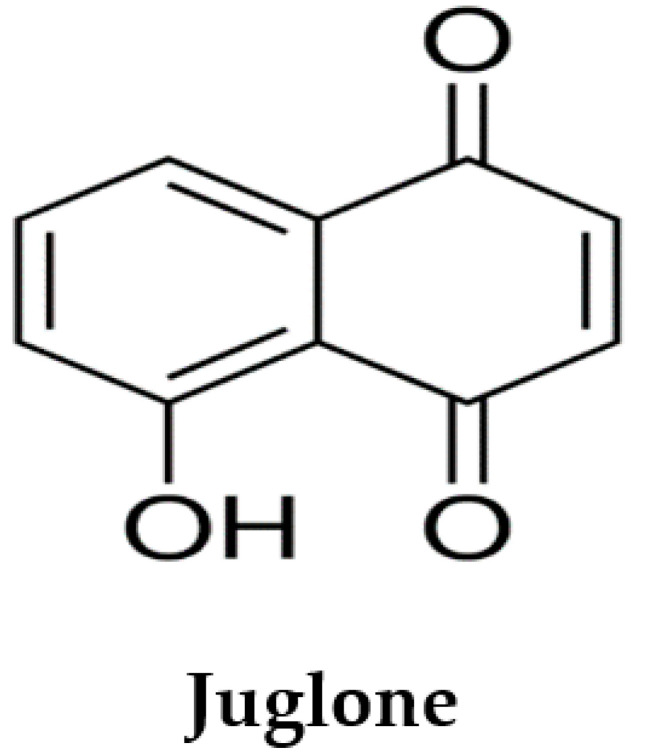
Chemical structure of juglone.

Juglone (5-hydroxy-1,4-naphthoquinone; Figure 1) is a phenolic compound from the black walnut tree (*Juglans nigra*) that has a wide range of properties considered beneficial to health. *Juglans regia* L extract is reported to possess antioxidant and cardioprotective effects against isoproterenol-induced myocardial infarction in rats [23,24]. In the 1850s, juglone was first isolated from the walnut tree [25] and in 1881, the first scientific report on the juglone allelopathic effect was published [26]. Juglone has been reported as allelopathic [26], vasorelaxant, antihypertensive [27,28,29,30], antifungal [31,32], antitumour [33,34,35], antibacterial [32,36] and antioxidant [23,28,37,38]. However, no study was found on the cardioprotective activity of juglone. Hence, the aim of our study was to examine the effects of juglone against isoproterenol-induced MI in rats.

## 2. Methods and Materials

### 2.1. Chemicals and Reagents

Standard drugs such as atenolol and isoproterenol were purchased from Sigma-Aldrich, St. Louis, MO, USA. Pentothal sodium and heparin injections were obtained from Abbot Laboratories, Karachi, Pakistan and F. Hoffmann-La Roche, Basel, Switzerland, respectively. All drugs were dissolved in distilled water/normal saline, except juglone. The juglone was first dissolved in dimethyl sulfoxide (DMSO) and then diluted with distilled water (the final in vivo study doses contain maximum 1% DMSO). 

### 2.2. Experimental Animals and Housing Conditions

The cardioprotective activity was conducted on 8–10 week-old Sprague Dawley (SD) rats (200–250 g) (preferably male), which were housed at the Animal House of the COMSATS University Islamabad, Abbottabad Campus, maintained at 23–25 °C. The experiments performed complied with the rulings of the Institute of Laboratory Animal Resources, Commission on Life Sciences, National Research Council [39] (NRC, 1996) and approved by the Ethical Committee of Department of Pharmacy, COMSATS University Islamabad, Abbottabad campus, in its meeting held on 17 June 2013, video notification EC/PHM/07-2013/CIIT/ATD.

### 2.3. Cardioprotective Study

#### 2.3.1. Cardioprotective Study of Juglone against the Isoproterenol-Induced MI in Rats

According to the literature [40,41,42] with a slight modification, the experimental myocardial infarction (MI) in rats was induced by administering isoproterenol (ISO) dissolved in normal saline and were administered at the dose of 80 mg/kg body weight subcutaneously (s.c.) at an interval of 24 h for 2 consecutive days. The test compound juglone at the doses of 1 and 3 mg kg/day were screened to determine the dose-dependent effect of test compounds in ISO-induced myocardial infarction in rats. 

The experimental animals were randomly divided into five groups consisting of six rats in each group. Group I (control group) rats received 1% DMSO (1 mL kg/day, i.p.) for 5 consecutive days and normal saline (1 mL/kg/day, s.c.) on the 4th and 5th day, at an interval of 24 h. Group II (ISO group) rats received 1% DMSO (1 mL/kg/ day, i.p.) for 5 consecutive days and ISO (80 mg kg^−1^, s.c.) on the 4th and 5th day, at an interval of 24 h. Group III (atenolol + ISO) rats received atenolol (1 mg/kg i.p.) for 5 consecutive days and then ISO (80 mg/kg, s.c.) on the 4th and 5th day, at an interval of 24 h. Moreover, in Group IV and V (juglone + ISO), juglone in two different doses (1 and 3 mg/kg i.p.) was administered via i.p. route for 5 consecutive days and then ISO (80 mg/kg, s.c.) on the 4th and 5th day, at an interval of 24 h. After 24 h of the 2nd dose of ISO (80 mg/kg, s.c.), rats were anesthetized with sodium thiopental (pentothal, 40–100 mg/ kg body weight). 

The body weight of all experimental rats was noted. An anesthetized rat was placed in a supine position. After complete induction of anesthesia, needle electrodes were inserted subcutaneously according to the lead II (left foreleg, right foreleg, and left rear leg) ECG scheme. ECGs were recorded using a PowerLab supplemented with an Animal BioAmp, analyzed by the LabChart 7 software (AD Instruments, Australia). After completion of ECG analysis, the rats were dissected and blood samples were collected through cardiac puncture. Hearts were removed, weighed, infarct areas were estimated and preserved in formalin solution (10%) for histopathology. The heart to body weight ratio of each animal was calculated to assess the degree of congestion [13]. The % change was calculated through the following formula:

$$\frac{Control-Change}{Change}\times 100$$

#### 2.3.2. Biochemical Estimations in Serum

The serums were separated by centrifugation for biochemical analyses. The cardiac marker enzymes, such as aspartate transaminase (AST), alanine transaminase (ALT), creatine phosphokinase (CPK), creatine kinase-MB (CK-MB), lactate dehydrogenase (LDH) and cardiac troponin I, were estimated using commercially available standard assay kits (Standbio Laboratory, Boerne, TX, USA; JAJ International Inc., San Diego, California, USA). The serum markers were estimated in the serum according to procedure followed by Kim et al. 2015 [13] and Ighodaro et al. 2018 [15].

#### 2.3.3. Histopathological Examination

After sacrifice, the heart tissues were rapidly dissected out and washed immediately with ice-cold normal saline and fixed in 10% buffered formalin. Then, the rats’ heart samples were dehydrated in graded (80–100%) alcohol and cleared in xylene. The fixed tissues were embedded in paraffin, 5 mm sections were prepared with the help of microtome and stained with hematoxylin (H) and eosin (E) stains for light microscopy. The H and E stains were used to impart colors to a specimen. The stained sections under a light microscope were examined for myocardial changes, such as changes in the branches of cardiac muscles fibers, inflammation, edema and necrosis. These slides were photographed through an advanced research microscope [40,43].

### 2.4. Statistical Analysis

Statistical analyses of the biochemical data were determined by using one way ANOVA followed by Tukey’s test through GraphPad Prism Software, version 8 (Graph Pad, San Diego, CA, USA). A *p* value of less than 0.05 (* *p* < 0.05) was considered statistically significant.

## 3. Results

### 3.1. Cardioprotective Effect of Juglone against the Isoproterenol (ISO)-Induced Myocardial Injury in Rats

#### 3.1.1. Effect of Juglone on Electrocardiograph (ECG) Parameters

**Figure 2:**
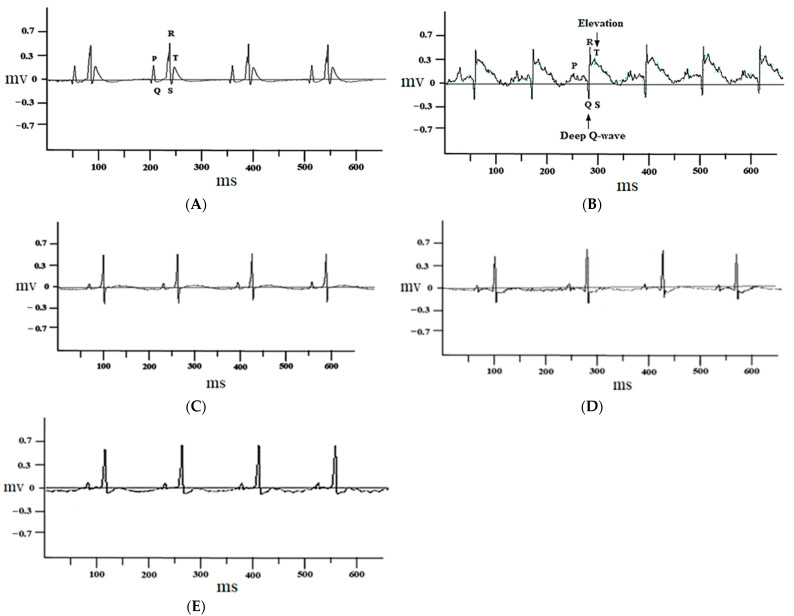
Representative electrocardiogram (ECG) tracing of control (**A**), isoproterenol (**B**), atenolol (1 mg/kg) + ISO (**C**), juglone 1 mg/kg + ISO (**D**) and 3 mg/kg + ISO (**E**) treated rats (recorded from lead II with recording speed 50 ms/div). Millisecond (ms); millivolt (mv).

The control group rats treated with 1% DMSO alone did not show any change in the electrocardiograph pattern (Figure 2A), while ISO-injected rats showed marked ST-segment elevation and deep Q waves (Figure 2B). These changes were restored to nearly normal with atenolol (1 mg/kg) (Figure 2C) and juglone (1 and 3 mg/kg) (Figure 2D,E) pretreatment, when compared with isoproterenol-alone administered rats (Figure 2B).

#### 3.1.2. Effect of Juglone on Heart and Body Weight Ratio

**Figure 3:**
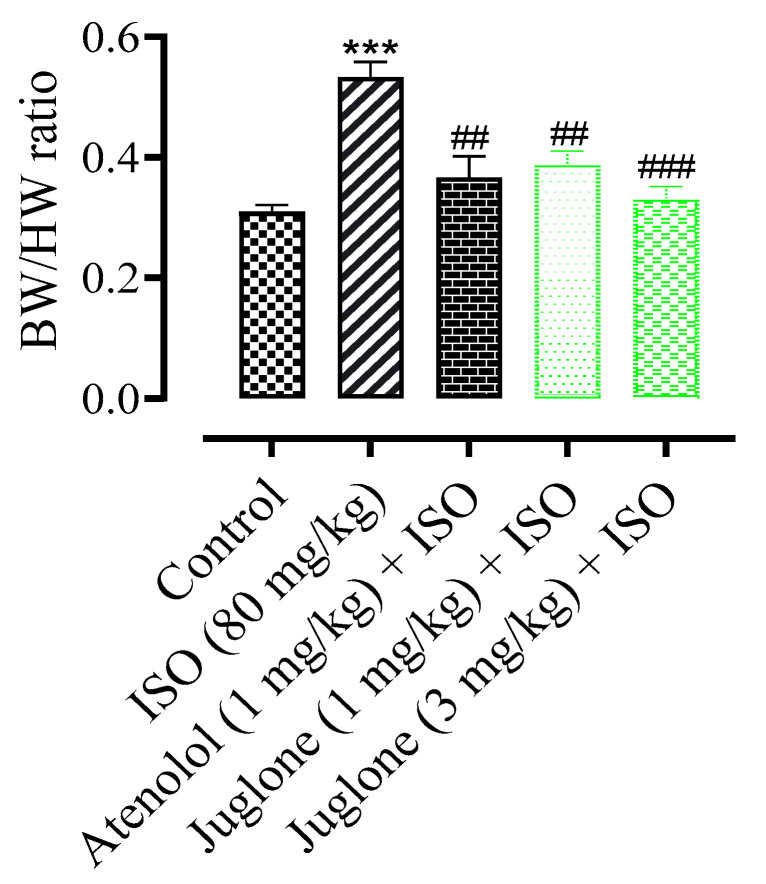
Effect of juglone + ISO, atenolol + ISO and ISO alone on body weight/heart weight (BW/HW) ratio in comparison to control. Values are expressed as mean ± SEM (n = 6), ehere ^##^ *p* < 0.01 vs. ISO, ^###^ *p* < 0.001 and *** *p* < 0.001 vs. control (one-way ANOVA followed by a Tukey’s test).

The mean body weight of rats at the end of experiment period in all experimental groups had no significant changes (data not shown). The heart weight and the ratio of heart weight to body weight were increased significantly (*p* < 0.001) in ISO-administered groups when compared with the normal control groups. The rats were pretreated rats, pre-co-treated with the atenolol and juglone (1 mg and 3 mg) and showed a significant reduction in heart weight and ratio as compared to the ISO-alone administered groups (Figure 3).

#### 3.1.3. Changes in the Rat Heart Structural Anatomy and Measurement of Myocardial Infarct Size

**Figure 4:**
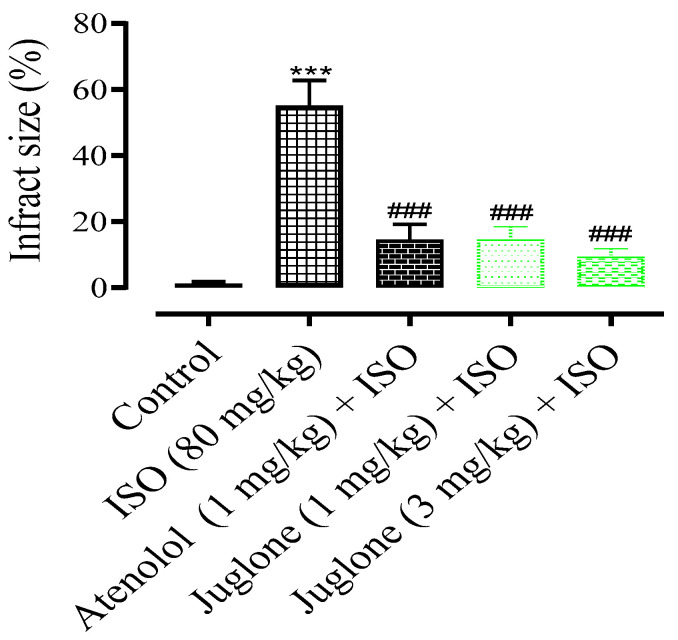
The graph shows the percent myocardial infarct size in the control and experimental animals. Values are expressed as mean ± SEM (n = 6), where ^###^ *p* < 0.001 vs. ISO and *** *p* < 0.001 vs. control (one-way ANOVA followed by a Tukey’s test).

In comparison to the control group, the rat heart shape changed from elliptical to spherical when treated with isoproterenol (80 mg) and the heart shape was significantly protected in the atenolol (1 mg) and juglone (1 mg/kg and 3 mg/kg) treated groups. The infarcted myocardium appeared pale grey or white, from which the percent infarct area was identified. The infarction was significantly prevented in the treated groups, including atenolol (15%) and juglone 1 mg (15%) and 3 mg (10%) as compared to isoproterenol (55%), as shown in Figure 4.

#### 3.1.4. Effect of Juglone on Cardiac Marker Enzymes

**Figure 5:**
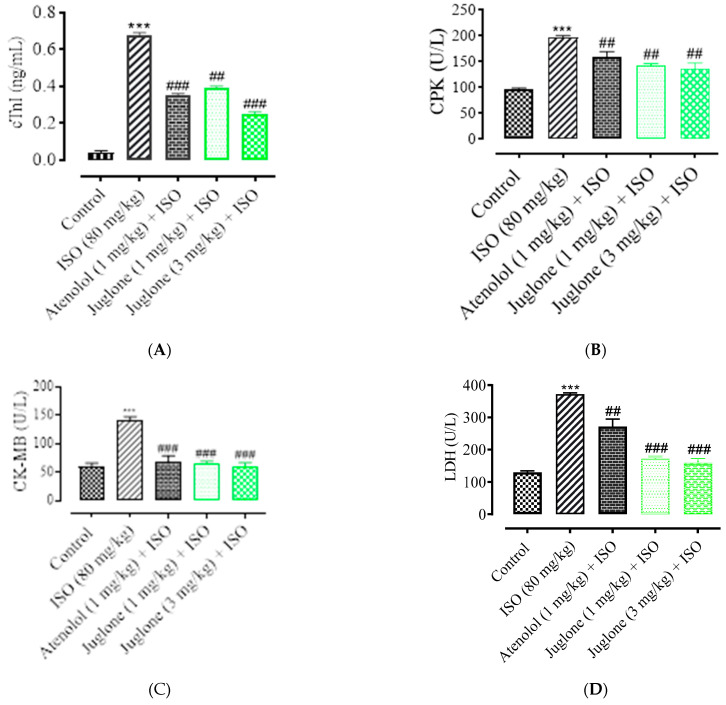

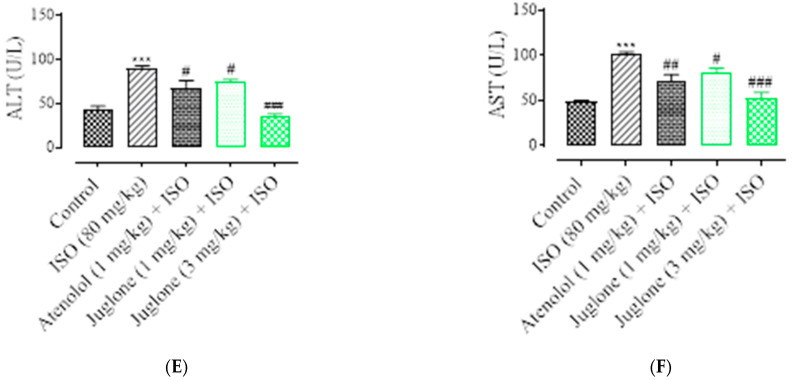
Effect of juglone and atenolol on cardiac troponin I (cTnI) (**A**) creatine phosphokinase (CPK) (**B**), creatine kinase-MB (CK-MB) (**C**), lactate dehydrogenase (LDH) (**D**), alanine transaminase (ALT) (**E**) and aspartate transaminase (AST) (**F**) in isoproterenol (ISO)-induced ischemic rats. Values are expressed as mean ± SEM (n = 6), where ## *p* < 0.01 vs. ISO, ### *p* < 0.001 vs. ISO and *** *p* < 0.001 vs. control (one-way ANOVA followed by a Tukey’s test). # *p* < 0.05 vs. ISO, ## *p* < 0.01 vs. ISO, ### *p* < 0.001 vs. ISO and *** *p* < 0.001 vs. control (one-way ANOVA followed by a Tukey’s test).

As shown in Figure 5, there was a significant elevation observed in the serum levels of diagnostic marker enzymes (cTnI, CPK, CK-MB, LDH, ALT and AST) in the serum of ISO-alone administered rats. Compared with the control, ISO alone caused a significant increase in the serum level of myocardial injury biomarkers, including cTnI (82.0 ± 3.01%), CPK (49.50 ± 4.01%), CK-MB (54.04 ± 2.01%), LDH (60.25% ± 5.01%), ALT (46.8 ± 3.02%) and AST (51.3 ± 2.02%). Pretreatment with atenolol (1 mg/kg) significantly restored the serum levels of the cardiac biomarker alterations induced by ISO, such as cTnI (48.52 ± 1.02%), CPK (24.75 ± 1.24%), CK-MB (53.0 ± 1.54%), LDH (27.30 ± 4.02%), ALT (24.13 ± 2.02%), AST (27.34 ± 1.91%). Pretreatment of juglone (1 mg/kg) significantly decreased the serum level of cTnI (42.64 ± 1.91%), CPK (28.12 ± 1.68%), CK-MB (54.36 ± 2.11%), LDH (54.36 ± 5.02%), ALT (18.14 ± 1.14%), AST (19.0 ± 2.05%). In addition, pretreatment with juglone (3 mg/kg) also significantly restored the serum level of the cardiac biomarker alterations induced by ISO, such as cTnI (63.23 ± 1.25%), CPK (32.22 ± 2.51%), CK-MB (58.35 ± 3.01%), LDH (58.85 ± 6.01%), ALT (59.01 ± 3.10%) and AST (48.01 ± 3.31%) as compared to the ISO-alone administered group (Figure 5A–F).

#### 3.1.5. Effect of Juglone on Histological Changes

**Figure 6:**
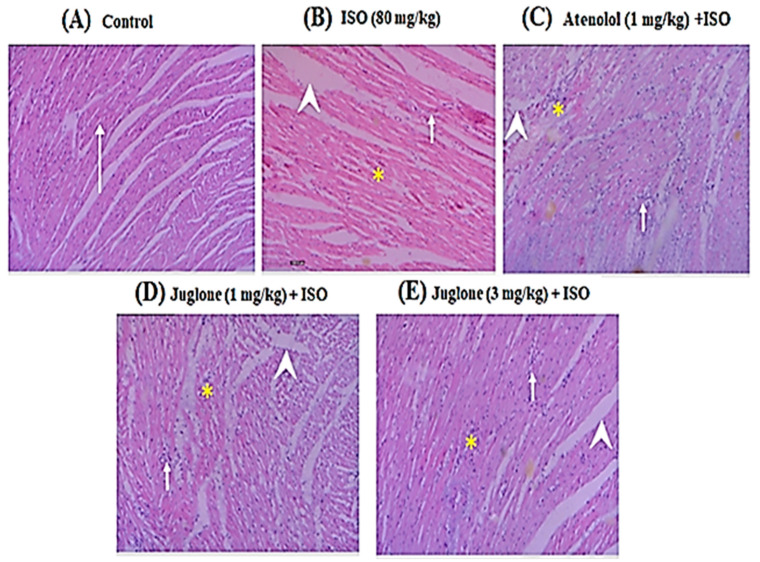
Heart tissue (H&E, 200×) of (**A**) control, shows normal cardiomyocytes (arrow), (**B**) isoproterenol (ISO)-alone treated shows marked inflammation (arrow), edema (arrow head) and confluent necrosis (asterisk), (**C**) pretreatment with atenolol + ISO produced moderate reduction in inflammation (arrow), edema (arrow head) and necrosis (asterisk), (**D**) 1 mg/kg juglone + ISO produced moderate reduction in inflammation (arrow), edema (arrow head) and necrosis (asterisk) and (**E**) 3 mg/kg juglone + ISO produced maximal reduction in inflammation (arrow), edema (arrow head), and necrosis (asterisk), respectively.

Histopathological observations of the control rat heart showed normal morphology of cardiac muscle fibers with striations and branched appearance (Figure 6A). ISO-induced rats revealed marked inflammation, edema and necrosis (Figure 6B). The tissue sections from all treated groups, atenolol (1 mg/kg) + ISO (Figure 6C) and juglone (1 and 3 mg/kg) + ISO (Figure 6D,E), showed less inflammation and edema and also the morphology of cardiac muscle fibers was relatively well preserved with minimum evidence of necrosis, when compared to ISO-induced group, as shown in Figure 6 and Table 1.

## 4. Discussion

To test juglone as a possible cardioprotective agent, an ISO-induced myocardial ischemic model in rats was developed. Furthermore, the plant source of juglone is reported to possess antioxidant and cardioprotective activities [24]. ISO is a synthetic catecholamine and non-selective beta-adrenergic receptor agonist. β-adrenergic receptor (β-AR) signaling is the primary mechanism to increase cardiac contractility. However, chronic β-AR stimulation, which occurs in MI, results in dysregulation of the β-adrenergic pathway, leading to activation of protein kinase A (PKA)-dependent phosphorylation of L-type calcium channels, which in response lead to an increase in the intracellular calcium level and oxidative stress [44,45]. A subcutaneous injection of a high dose of isoproterenol caused severe stress in the myocardium, resulting in necrosis of the heart muscles, elevated ST-segments and deep Q-waves in the ECG and structural changes in the heart very similar to that which occurs in patients with MI [46]. Increased heart rate leads to increase oxygen consumption and accelerates myocardial necrosis [40]. Despite some differences, there are essential similarities between rat and human ECGs [47]. Rat myocardial changes induced by ISO are almost similar to human myocardium changes during MI [48].

The ECG is considered one of most important initial clinical markers for diagnosing MI. Its correct interpretation is usually the basis for immediate therapeutic interventions and/or subsequent diagnostic tests. ECG abnormalities, such as ST-segment elevation and deep Q-waves, could be due to the successive loss of cell membrane and absence of electrical activity in the injured myocardium [49]. ST-elevation myocardial infarction (STEMI) is an event in which transmural myocardial ischemia results in myocardial injury or necrosis [50]. Pretreatment of ISO-treated rats with different doses of juglone and standard drugs, such as atenolol, markedly restrained ISO-induced changes in the ECG pattern, ST-segment elevation and deep Q-waves, suggesting membrane protecting effects. Juglone highly significantly restored the ECG pattern at maximum doses (3 mg/kg). The cardioprotective action of juglone was further confirmed. 

The myocardium contains an abundant amount of diagnostic marker enzymes for MI and once metabolically damaged, these contents release into the extracellular fluid [51]. According to the 2018 clinical definition, MI confirmation requires the validation of the cardiac injury with abnormal cardiac biomarkers [52]. The ISO administration to the rats also caused the leakage of cardiac marker enzymes, such as cTnI, CPK, CK-MB, LDH, AST and ALT, which may be due to myocardial necrosis, damage or destruction of cell membrane, deficiency of oxygen (hypoxia) or glucose. Moreover, an increase in the heart to body weight ratio with abnormal histo-architecture of heart tissue indicated ISO-induced myocardial damage, which was in line with the previous reports [53,54,55,56]. 

Pretreatment with juglone (1 and 3 mg/kg, respectively) and the standard drug atenolol, followed by ISO administration, significantly (*p* < 0.001) decreased the difference in the heart to body weight ratio and infarct size when compared to ISO-alone treated rats. The infarct area observed in rats pretreated with juglone was also reduced significantly (10%). Furthermore, juglone also significantly decreased (63%) the serum levels of cTnI. Cardiac troponin is the gold standard for diagnosing a MI. The three-subunits of troponin complex troponin C (TnC), troponin T (TnT) and troponin I (TnI), along with tropomyosin, are located on the actin filament and are crucial for the calcium-mediated regulation of cardiac muscle contraction [57,58]. cTnI and cTnT, which are specific to cardiac muscles, are the most common indicators for the diagnosis of heart damage after MI. cTnI is more sensitive to cardiac injury as compared to cTnT [59]. Troponin levels become elevated within 2 to 3 hrs after the onset of MI, reaching a peak at 18–24 h, and remain elevated for up to 14 days. The half-life of circulating troponin is nearly 2 h. So, it stays in the bloodstream for days after all the other biomarkers have returned to normal levels [49].

The appearance of CPK in the blood has been generally considered to be an indirect marker of cardiac muscle damage. The significant decrease (32%) in the serum level of CPK was observed with juglone. Juglone also produces a highly significant decrease (58%) in the serum levels of CK-MB. CPK is an enzyme that catalyzes the phosphorylation of creatine. By utilizing adenosine triphosphate, CPK converts creatine to phosphocreatine and adenosine diphosphate. CPK can be measured several times over a 24 hr period [60,61]. CK is found in both the cytosol and mitochondria of tissues. The cytosolic CK is composed of two types of subunit, M (muscle type) and B (brain type). These subunits allow the formation of three tissue-specific isoenzymes, CK-MB (cardiac muscle), CK-MM (skeletal muscle) and CK-BB (brain). CK-MB is more sensitive to heart damage following MI. CK-MB is detectable in the blood within 3 to 5 hrs after a MI, rises to a peak within 12-24 hrs, and is back to normal within a day or two [62,63]. 

The high level of LDH concentrations in the blood is indicative of tissue damage and inflammatory changes, particularly in the heart [64]. Juglone significantly reduced (58%) the serum level of LDH. LDH is an enzyme found in almost every cell that mainly presents in the myocardium, skeletal muscle, liver and kidneys. In the heart, LDH is present in both mitochondria and surrounding cytosol and organelles. The LDH turns sugar into energy [65]. The LDH isoenzyme level increases 24–72 h following MI and reaches its peak concentration in 3–4 days. The levels remain elevated for 8 to 14 days. LDH is considered a non-specific marker for MI [16].

The concentrations of AST and ALT in the serum at normal physiological condition are low. However, the level of these markers increases following MI, causing leakage of AST and ALT into the blood circulation. The juglone highly significantly decreases (59%, 48%) the serum level of these enzymes. ALT is mainly located in the cytoplasm, while AST is present both in the cytoplasm and mitochondria, which is why AST is more sensitive to cardiac injury as compared to ALT. After MI, AST and ALT are elevated at 6 to 10 hrs, peak at 24–36 hrs, then remain high for 3–5 days. In addition to ALT and AST in diagnosing MI, they have been replaced by newer enzymes and proteins that are more specific for cardiac damage, such as cTnI, CPK, CK-MB and LDH [66,67]. It has been demonstrated that juglone could maintain cardiac membrane structural and functional integrity and/or permeability, thereby restricting the leakage of the cardiac biomarkers from the myocardium. These effects are evident from the markedly blunted levels of cardiac biomarkers in juglone + ISO groups when compared to the ISO-alone treatment group, thereby establishing the cardioprotective effects of juglone.

Furthermore, the preliminary histopathological findings of the ISO-induced MI group myocardium showed large infarcted zones with inflammation, edema, coagulative necrosis and separation of cardiac muscle fibers. In rats, ISO had been reported to increase oxygen demand, deplete ATP levels, cause calcium overload and undergo autoxidation, leading to the formation of free radicals [68]. However, juglone and atenolol pretreatment followed by ISO considerably prevented the pathological changes, such as inflammation, edema and necrosis, when compared to ISO-alone treated rats. Previous studies found that juglone showed significant free radical scavenging activity [38,69]. The structure of juglone (Figure 1) contains hydroxyl groups in the benzene ring, which may explain its ability to scavenge free radicals [70]. Moreover, the plant source of juglone, *Juglans regia* extract, improved the activities of superoxide dismutase (SOD) and catalase (CAT) by scavenging superoxide and hydrogen peroxides produced by ISO [24]. Administration of submaximal doses of ISO have been reported to induce severe oxidative stress [71,72]. The generation of ROS occurs by the leakage of electrons into oxygen from various systems. Overproduction of ROS can cause severe impairment of cellular functions and necrotic lesions in the myocardium of rats. In contrast, superoxide dismutase (SOD), catalase (CAT) and glutathione peroxidase (GPX) constitute a mutually supportive and free radical scavenging enzymes team of first line defense against oxidative injury [73]. The plant source of juglone, *Juglans regia,* is also reported to inhibit the increase in intracellular malondialdehyde (MDA) levels [74]. MDA and its measurement have been used as indicators of lipid peroxidation [75]. Lipid peroxidation is considered a major mechanism of oxygen free radical toxicity [76]. Zhou et al. (2015) [76] demonstrated that juglone increased the activity of SOD and decreased oxidative stress. However, further studies have been suggested related to the effect of juglone on CAT and GPx. Moreover, juglone also activates mitogen-activated protein kinases (MAPK) that could promote cell survival, thereby protecting against conditions such as cardiac injury [35]. These reported activities of juglone suggest that it might be possible that their antioxidant effect is cardioprotective.

These primary findings of our study indicate the cardioprotective effect of juglone. The most significant effects were observed at higher doses (3 mg/kg). Moreover, juglone was found to be safe up to 3 mg/kg and the toxic dose observed in rats was 6 mg/kg (in the initial screening). In summary, this experimental evidence might be helpful to understand the beneficial effects of juglone against myocardial injury, although further studies are needed to confirm its mechanisms. 

## 5. Conclusions

In conclusion, the results of our present study suggest that prior administration of juglone proved to be effective as a cardioprotective therapeutic agent in reducing the level of myocardial damage (induced by ISO) through fortifying the myocardial cell membrane, in a similar way to atenolol. Further electrophysiological studies and protein expression of different inflammatory mediators, such as caspase-3 and 9 etc., would provide more insight into the molecular aspects of this study.

## Figures and Tables

**Table 1:** Preventive effects of juglone and atenolol on the degree of histological changes in myocardium of isoproterenol (ISO)-administered ischemic rats.

Groups	Inflammation	Edema	Necrosis
Control	-	-	-
ISO (80 mg/kg)	+++	+++	+++
Atenolol (1 mg/kg) + ISO	+	+	+
Juglone (1 mg/kg) + ISO	+	++	+
Juglone (3 mg/kg) + ISO	+	+	-

The signs indicate: -, absent; +, mild changes; ++ moderate changes; and +++, marked changes, ISO; isoproterenol.

## Data Availability

The datasets used and analyzed during the current study are available from the corresponding author upon reasonable request.
